# Water balloon-induced orbital fracture in an aviator

**DOI:** 10.1186/s40779-019-0210-0

**Published:** 2019-06-11

**Authors:** Timothy E. Holland, David M. Smith, Guy N. Gibson, Jared G. Brinkerhoff

**Affiliations:** 1 Flight and Operational Medical Clinic, 78th Medical Group, 655 Seventh Street, Robins Air Force Base, Robins, GA 31098 USA; 2 Department of Radiology, Ehrling Bergquist Clinic, 2501 Capehart Rd, Offutt AFB, NE, Offutt, 68113-2160 USA; 3 Flight and Operational Medical Clinic, 5th Medical Group, 194 Missilie Ave, Minot AFB, ND, Minot, 58705 USA

**Keywords:** Boyle’s law, Orbital emphysema, Facial fracture

## Abstract

**Background:**

Orbital fractures are common injuries found in facial trauma. Typical etiologies of orbital fractures include motor vehicle collisions and assault. We report the case of a 32-year-old male who suffered an orbital fracture from a water balloon. Additionally, we describe the aeromedical complications that may result from this injury. Finally, we attempt to answer the question of when a patient may return to flying after sustaining such an injury through review of the literature.

**Case presentation:**

A 32-year-old male pilot with the United States Air Force was at an outdoor event with his unit when he was struck with a water balloon launched from a sling shot into his left orbit. Shortly afterwards, he had an onset of subcutaneous emphysema and was escorted to a nearby Emergency Department. Computed tomography identified an orbital fracture with associated orbital and subcutaneous emphysema. The patient was evaluated by a plastic surgeon and was determined not to be a surgical candidate. Four weeks later, he returned to flying status.

**Conclusions:**

Water balloons are thought to be safe and harmless toys. However, when coupled with slingshots, water balloons can become formidable projectiles capable of significant orbital injury including orbital fractures. These injuries are concerning to aviators, as the most common sites for fractures of the orbit are the thin ethmoid and maxillary bones adjacent to the sinuses. At altitude, gases in the sinuses may expand and enter the orbit through these fractures, which may suddenly incapacitate the flyer. It is important for flight surgeons to identify and assess these individuals to determine suitability for flying.

## Background

Orbital fractures are relatively common sequelae found in patients who have sustained blunt facial trauma; an estimated 10% of all facial fractures involve the orbit [1]. The thinner orbital floor and medial wall are the most common sites of fracture [1]. Typical etiologies of orbital fractures include motor vehicle collisions, assault, and sports-related injuries [2, 3]. Although often managed conservatively, approximately 25% of these fractures require operative treatment to prevent complications such as enophthalmos, orbital emphysema, extraocular muscle entrapment, and degradation/loss of vision [4]. With patients whose occupations involve regular flying and exposure to the aerospace environment (such as pilots), determining when it is appropriate for these personnel to return to their duties presents a challenge. Fractures extending into the adjacent sinuses may present an abnormal communication of air, which could be problematic at higher altitudes.

Perhaps more important is the mechanism of injury, or rather, the projectile causing the fracture. How exactly does a malleable, water-filled, small piece of latex induce an injury usually claimed by fists and automotive collisions?

### Case presentation

**Fig. 1:**
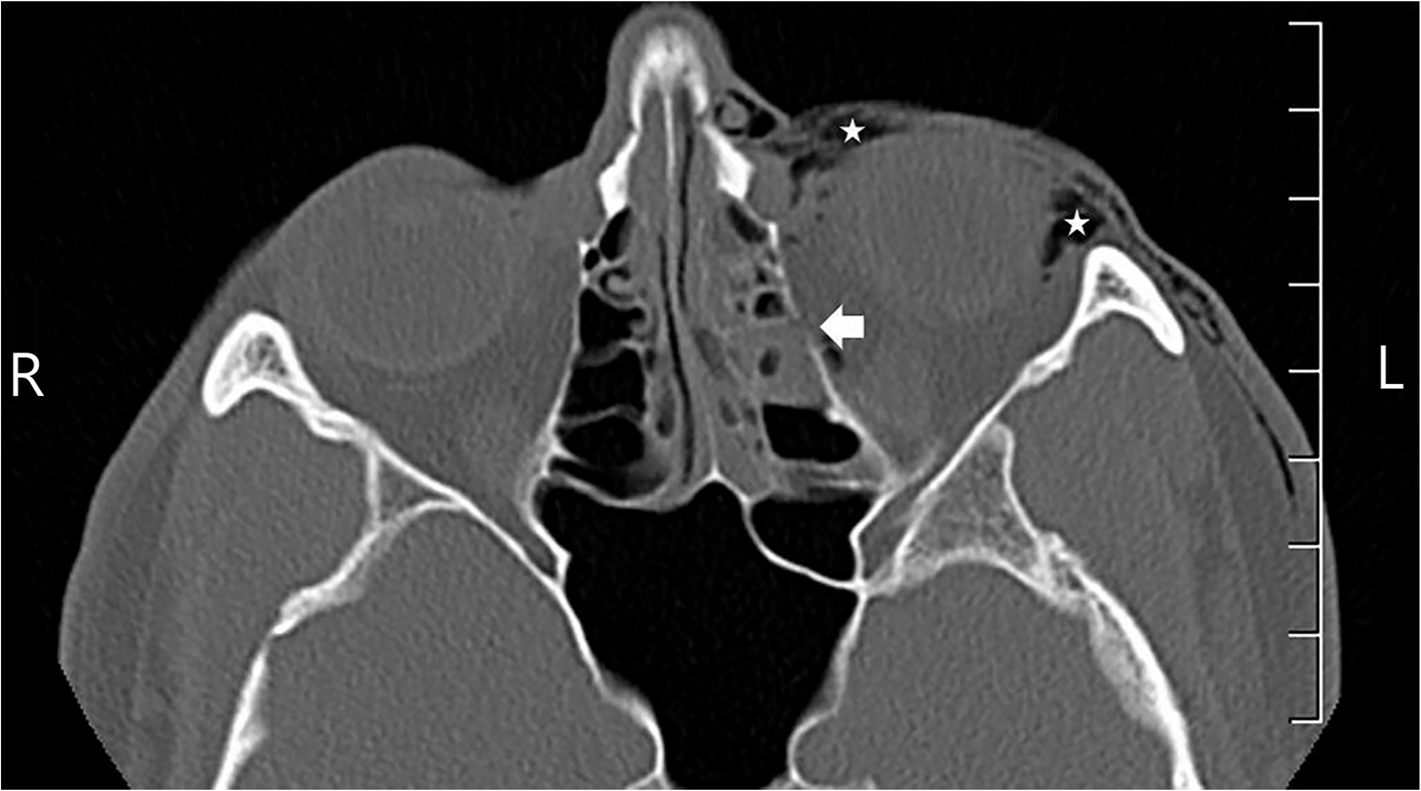
Axial noncontrast CT of the orbits. Axial CT of the orbits demonstrates a 5-mm nondisplaced fracture through the medial wall of the left orbit, which results in communication with the adjacent ethmoid air cells (thick arrow). Subcutaneous emphysema is present within the pre and postseptal regions and anterior soft tissues (starred). A single locule of air was noted within the intraconal left orbit

**Fig. 2:**
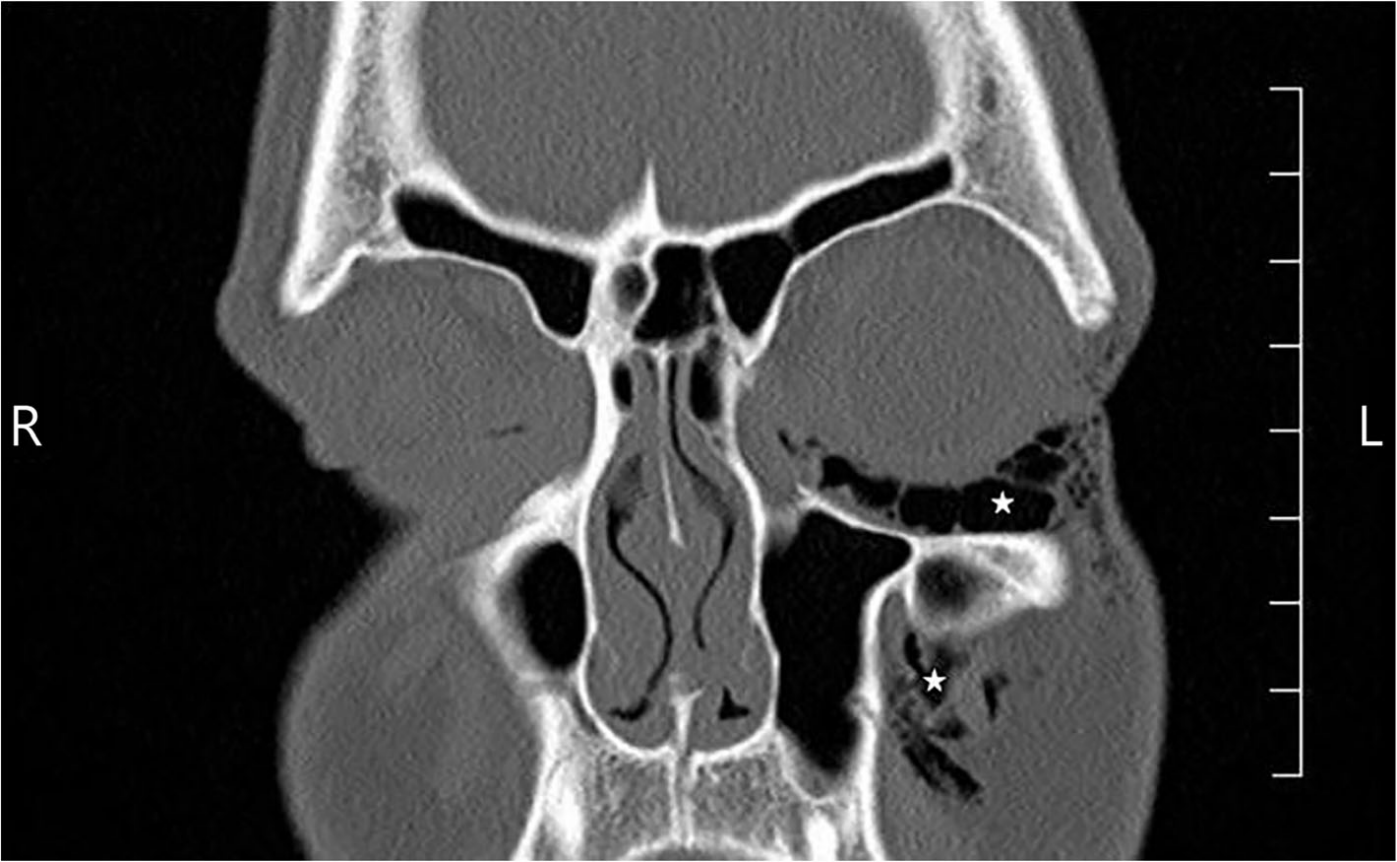
Coronal noncontrast CT of the orbits. Coronal CT of the orbits demonstrates the extent of subcutaneous emphysema within the intraorbital and periorbital soft tissues (starred)

The patient is a 32-year-old male without any significant past medical history. He is an active duty United States Air Force (USAF) Joint Surveillance Target Attack Radar System (JSTARS) pilot who was at an informal, outdoor military function with his unit when he was struck with a water balloon launched by a slingshot into his left eye. The patient was not wearing any glasses or eye protection. Two physicians were on scene and immediately evaluated him in the field. On presentation, the patient complained of blurry vision, mild left eye pain, and a bloody nose. He denied any double vision. Physical exam was significant for periorbital swelling, mild injection of the sclera, and moderate epistaxis. Visual fields were grossly assessed and within normal limits. All extraocular movements were intact despite mild pain on left lateral gaze. The pupils were equal, round, and reactive, and there was not complete 360° subconjunctival hemorrhage. The patient was asked if he experienced any changes in vision, which he denied. During our examination, the patient attempted to clear some of his epistaxis by blowing his nose, and he immediately developed subcutaneous emphysema with increased pain in his left eye. He was escorted to the nearby emergency department for further evaluation. Computed tomography (CT) of his orbits demonstrated a nondisplaced left medial orbital wall fracture with orbital and subcutaneous emphysema (Figs. 1 and 2). The patient was administered intravenous ampicillin/sulbactam and transferred to another hospital for evaluation by a plastic surgeon.

The plastic surgeon determined he was not a surgical candidate, stating the patient’s fracture was nondisplaced and without other serious comorbidities, such as exophthalmos or extraocular muscle entrapment. Our patient was then discharged. On follow-up the next week, the surgeon recommended conservative treatment and that the patient be ‘cleared for full duty and flight status.’ Follow-up with an optometrist noted no significant ocular trauma and no change in visual acuity. Finally, a flight surgeon evaluated him at the patient’s duty station. After thorough examination noting complete resolution of the patient’s symptoms and with the concurrence of the plastic surgeon, the patient was returned to flying status 4 weeks after his initial injury.

We followed up with the patient several months later to discuss his injury and its relation to his flying career. Fortunately, he reported no further symptoms on the ground or in the air, and he has had no other instances of subcutaneous or orbital emphysema.

## Discussion and conclusions

We report this case for two reasons: the unusual culprit of the patient’s injury and the aeromedical concerns of an orbital fracture with communication into the ethmoid sinus.

Water balloons are generally thought to be safe and harmless. However, when coupled with a slingshot, these same water balloons become formidable projectiles capable of significant injury. Several case reports were found in a literature review that demonstrates the following. The first report by Holds et al. [5] in 1988 involved a 23-year-old male who was struck by a water balloon fired from a slingshot located 300 m away. His injury resulted in concussion, vitreous hemorrhage, retinal edema, enophthalmos, large orbital blowout fractures, and inferior and medial rectus muscle entrapment. On long-term follow-up, the patient had an afferent pupillary defect, macular hole, and visual acuity limited to 20/300. Another case report demonstrated significant injury from a slingshot propelled water balloon leading to retinal detachment [6].

Other water balloon injuries were discussed in a study by Bullock et al. [7]. The authors described ophthalmic injuries from 17 patients with injuries ranging from periorbital edema and ecchymosis to retinal hemorrhages and optic atrophy. Their report also contained an experimental study where the kinetic energies of water balloons were measured after being fired from a commercially available slingshot. Maximum velocities launched by this slingshot reached speeds of 152–245 km/h, yielding kinetic energies ranging from 141 to 232 joules. To put this into perspective, a 22-caliber round fired from a small rifle at approximately 1238 km/h yields a kinetic energy of 170 joules. The authors demonstrated the significance of this kinetic energy by launching a 100-g water balloon at a 12-kg watermelon. The watermelon subsequently exploded as a result of 240 joules of energy or the equivalent of dropping that same watermelon from a height of 2 m onto a hard surface.

As our patient is an active duty USAF pilot, he is expected to consistently expose himself to the aeromedical environment. An orbital fracture with communication to the sinuses presents an issue at altitude. Boyle’s law states that the volume and pressure of a gas are inversely proportional at a constant temperature. Therefore, as an aircraft ascends and the cabin pressure decreases from its pressure at sea level, enclosed gases will expand. If there is a communication between the sinuses and an orbit, as noted in our patient, these gases could potentially cause orbital emphysema and orbital compartment syndrome. If experienced by the pilot, it would present a risk to not only the patient but to the safety of the flight and all passengers on board. It would be prudent in this circumstance to have the pilot grounded until the condition resolves.

Potential complications of air travel were evaluated in a retrospective study by Tan-Gore et al. [8]. The authors described 48 patients in Northern Australia with orbital fractures who were transported via airlift to a hospital for more definitive care. The records of these patients were reviewed to discover any documented evidence of complications arising from air transport. Surprisingly, the authors did not uncover any documentation of adverse events from aeromedical transport. Although reassuring, this does not exclude the possibility of developing complications. Other case studies in the literature describe instances where patients later developed symptoms of orbital emphysema after either air travel or nose blowing [9, 10]. Perhaps in those case reports, the external pressure changes may have caused sufficient force to fracture a previously weakened, thin-walled orbit.

Unfortunately, the literature is not clear on length of time to refrain from air travel for patients with orbital fractures. Since there is a heightened concern for complications with a pilot, it is important to arrive at a more definitive answer to when a return to flying is considered appropriate and safe. A study by Mahmood et al. [11] attempted to answer this question by surveying 184 oral and maxillofacial surgeons (OMFS) in the UK and asking them how long they recommend patients to refrain from nose blowing or travel by airplane after suffering zygomatic complex fractures. Their results showed a wide variation: 40% of OMFS made no recommendations, 30% recommended avoiding air travel for 8 to 14 days, and 15% for 3 to 8 weeks. Of note, most of the OMFS based these recommendations on ‘traditional practice and common sense’.

A review of bone physiology and fracture healing offers another perspective to the studies mentioned above. Fractures are considered completely healed when there is presence of lamellar or mature bone and excessive callus is resorbed. This process begins approximately 6 weeks after injury, and removal and reorganization of repair tissues may continue for several years. However, bone callus may be observed replacing cartilage approximately 2 to 3 weeks after the initial fracture [12]. Although difficult to definitively conclude that bone callus at this stage possesses sufficient structural integrity, it does reinforce recommendations by some of the OMFS in the Mahmood et al. study [11].

Based on the reviewed literature and without any other significant variables or complications, we believe it is reasonable for a patient with a small, nondisplaced orbital fracture to be permitted to fly on aircraft approximately 4 weeks after injury. This recommendation should be used cautiously, and proper clinical judgment should be used to make an appropriate aeromedical disposition on a case-by-case basis. Other facial or orbital fractures that are more extensive or require surgery would necessitate a different approach. Those patients may benefit from a longer period of abstinence from flying. Perhaps those who also serve as aircrew should additionally undergo altitude chamber training to assess the stability of their injuries.

## Abbreviations

CT: Computed tomography
JSTARS: Joint Surveillance Target and Attack Radar System
OMFS: Oral and maxillofacial surgeon
USAF: United States Air Force
